# Supplementary material for: A fault-intrusion-tolerant system and deadline-aware algorithm for scheduling scientific workflow in the cloud
Source: PeerJ Comput Sci. 2021 Nov 2;7:e747. doi: 10.7717/peerj-cs.747 (PMC8576554; doi:10.7717/peerj-cs.747)
Supplement: Supplemental Information 6 [file peerj-cs-07-747-s006.zip › Myproject1.1/docs/org/cloudbus/cloudsim/network/datacenter/NetworkDatacenter.html]

NetworkDatacenter


JavaScript is disabled on your browser.


- Overview
- Package
- Class
- Use
- Tree
- Deprecated
- Index
- Help

- Prev Class
- Next Class

- Frames
- No Frames

- All Classes

- Summary:
- Nested |
- Field |
- Constr |
- Method

- Detail:
- Field |
- Constr |
- Method


org.cloudbus.cloudsim.network.datacenter

## Class NetworkDatacenter

- java.lang.Object
- - org.cloudbus.cloudsim.core.SimEntity
  - - org.cloudbus.cloudsim.Datacenter
    - - org.cloudbus.cloudsim.network.datacenter.NetworkDatacenter

- All Implemented Interfaces:
  :   java.lang.Cloneable

  ---

    

  ```
  public class NetworkDatacenter
  extends Datacenter
  ```

  NetworkDatacenter class is a Datacenter whose hostList are virtualized and networked. It contains
  all the information about internal network. For example, which VM is connected to Switch etc. It
  deals with processing of VM queries (i.e., handling of VMs) instead of processing
  Cloudlet-related queries. So, even though an AllocPolicy will be instantiated (in the init()
  method of the superclass, it will not be used, as processing of cloudlets are handled by the
  CloudletScheduler and processing of VirtualMachines are handled by the VmAllocationPolicy.
  Please refer to following publication for more details:
  Saurabh Kumar Garg and Rajkumar Buyya, NetworkCloudSim: Modelling Parallel Applications in Cloud
  Simulations, Proceedings of the 4th IEEE/ACM International Conference on Utility and Cloud
  Computing (UCC 2011, IEEE CS Press, USA), Melbourne, Australia, December 5-7, 2011.

  Since:
  :   CloudSim Toolkit 3.0

- - ### Field Summary

    Fields

    | Modifier and Type | Field and Description |
    | --- | --- |
    | `java.util.Map<java.lang.Integer,java.lang.Integer>` | `HostToSwitchid` |
    | `java.util.Map<java.lang.Integer,Switch>` | `Switchlist` |
    | `java.util.Map<java.lang.Integer,java.lang.Integer>` | `VmtoHostlist` |
    | `java.util.Map<java.lang.Integer,java.lang.Integer>` | `VmToSwitchid` |

    - ### Fields inherited from class org.cloudbus.cloudsim.core.SimEntity

      `FINISHED, HOLDING, RUNNABLE, WAITING`
  - ### Constructor Summary

    Constructors

    Constructor and Description

    `NetworkDatacenter(java.lang.String name,
    DatacenterCharacteristics characteristics,
    VmAllocationPolicy vmAllocationPolicy,
    java.util.List<Storage> storageList,
    double schedulingInterval)`

    Allocates a new NetworkDatacenter object.
  - ### Method Summary

    Methods

    | Modifier and Type | Method and Description |
    | --- | --- |
    | `java.util.Map<java.lang.Integer,Switch>` | `getEdgeSwitch()` Get list of all EdgeSwitches in the Datacenter network One can design similar functions for other type of switches. |
    | `protected void` | `processCloudletSubmit(SimEvent ev, boolean ack)` Processes a Cloudlet submission. |
    | `boolean` | `processVmCreateNetwork(Vm vm)` Create the VM within the NetworkDatacenter. |

    - ### Methods inherited from class org.cloudbus.cloudsim.Datacenter

      `addFile, checkCloudletCompletion, contains, contains, getCharacteristics, getHostList, getLastProcessTime, getRegionalCisName, getSchedulingInterval, getStorageList, getVmAllocationPolicy, getVmList, predictFileTransferTime, processCloudlet, processCloudletCancel, processCloudletMove, processCloudletPause, processCloudletResume, processCloudletStatus, processDataAdd, processDataDelete, processEvent, processOtherEvent, processPingRequest, processVmCreate, processVmDestroy, processVmMigrate, registerOtherEntity, setCharacteristics, setLastProcessTime, setRegionalCisName, setSchedulingInterval, setStorageList, setVmAllocationPolicy, setVmList, shutdownEntity, startEntity, updateCloudletProcessing`
    - ### Methods inherited from class org.cloudbus.cloudsim.core.SimEntity

      `cancelEvent, clone, getEventBuffer, getId, getName, getNextEvent, getNextEvent, getState, numEventsWaiting, numEventsWaiting, pause, run, schedule, schedule, schedule, schedule, scheduleFirst, scheduleFirst, scheduleFirst, scheduleFirst, scheduleFirstNow, scheduleFirstNow, scheduleFirstNow, scheduleFirstNow, scheduleNow, scheduleNow, scheduleNow, scheduleNow, selectEvent, send, send, send, send, sendNow, sendNow, sendNow, sendNow, setEventBuffer, setId, setState, waitForEvent`
    - ### Methods inherited from class java.lang.Object

      `equals, finalize, getClass, hashCode, notify, notifyAll, toString, wait, wait, wait`

- - ### Field Detail


    - #### VmToSwitchid

      ```
      public java.util.Map<java.lang.Integer,java.lang.Integer> VmToSwitchid
      ```


    - #### HostToSwitchid

      ```
      public java.util.Map<java.lang.Integer,java.lang.Integer> HostToSwitchid
      ```


    - #### Switchlist

      ```
      public java.util.Map<java.lang.Integer,Switch> Switchlist
      ```


    - #### VmtoHostlist

      ```
      public java.util.Map<java.lang.Integer,java.lang.Integer> VmtoHostlist
      ```
  - ### Constructor Detail


    - #### NetworkDatacenter

      ```
      public NetworkDatacenter(java.lang.String name,
                       DatacenterCharacteristics characteristics,
                       VmAllocationPolicy vmAllocationPolicy,
                       java.util.List<Storage> storageList,
                       double schedulingInterval)
                        throws java.lang.Exception
      ```

      Allocates a new NetworkDatacenter object.

      Parameters:
      :   `name` - the name to be associated with this entity (as required by Sim\_entity class from
          simjava package)
      :   `characteristics` - an object of DatacenterCharacteristics
      :   `storageList` - a LinkedList of storage elements, for data simulation
      :   `vmAllocationPolicy` - the vmAllocationPolicy

      Throws:
      :   `java.lang.Exception` - This happens when one of the following scenarios occur:
          - creating this entity before initializing CloudSim package- this entity name is null or empty- this entity has zero number of PEs (Processing Elements).   
                No PEs mean the Cloudlets can't be processed. A CloudResource must contain one or
                more Machines. A Machine must contain one or more PEs.
  - ### Method Detail


    - #### getEdgeSwitch

      ```
      public java.util.Map<java.lang.Integer,Switch> getEdgeSwitch()
      ```

      Get list of all EdgeSwitches in the Datacenter network One can design similar functions for
      other type of switches.


    - #### processVmCreateNetwork

      ```
      public boolean processVmCreateNetwork(Vm vm)
      ```

      Create the VM within the NetworkDatacenter. It can be directly accessed by Datacenter Broker
      which manage allocation of Cloudlets.


    - #### processCloudletSubmit

      ```
      protected void processCloudletSubmit(SimEvent ev,
                               boolean ack)
      ```

      Processes a Cloudlet submission.

      **Overrides:**
      :   `processCloudletSubmit` in class `Datacenter`

      Parameters:
      :   `ev` - a SimEvent object
      :   `ack` - an acknowledgement


- Overview
- Package
- Class
- Use
- Tree
- Deprecated
- Index
- Help

- Prev Class
- Next Class

- Frames
- No Frames

- All Classes

- Summary:
- Nested |
- Field |
- Constr |
- Method

- Detail:
- Field |
- Constr |
- Method
